# Construct optimization for AAV-mediated human α-syn overexpression, and validation across research settings: Development of a shared tool for the research community

**DOI:** 10.1177/1877718X261447105

**Published:** 2026-05-25

**Authors:** Joaquin Pardo, Janitha Mudannayake, Luis Daniel Bernal-Conde, Ivette M. Sandoval, Ryan B. Dong, Martino Avallone, David J. Marmion, Addison Gralen, Lauren Waite, Andreas Heuer, Marcus Davidsson, Nicole K. Polinski, Tomas Björklund, Fredric P. Manfredsson

**Affiliations:** 1 Lund University, Molecular Neuromodulation, Wallenberg Neuroscience Center, Lund, Sweden; 2 Instituto de Investigaciones Bioquímicas de La Plata “Prof. Dr Rodolfo R. Brenner” (INIBIOLP), Consejo Nacional de Investigaciones Científicas y Técnicas (CONICET) – Universidad Nacional de La Plata (UNLP), 60 y 120, 1900 La Plata, Argentina; 3 Department of Translational Neuroscience, Barrow Neurological Institute, Phoenix AZ, USA; 4 Lund University, Behavioural Neuroscience Laboratory, Wallenberg Neuroscience Center, Department of Experimental Medical Sciences, Lund, Sweden; 5 The Michael J. Fox Foundation for Parkinson's Research, New York, NY, USA

**Keywords:** alpha-synuclein, α-syn, AAV, neurodegeneration, animal model, substantia nigra

## Abstract

The adeno-associated virus (AAV) alpha-synuclein (α-syn) overexpression model of Parkinson's disease (PD) shares many etiopathological features of the human disease, including the formation of aggregated α-syn, neuroinflammation, and nigral neurodegeneration. However, despite the high face validity of this model, it is notoriously variable, yielding inconsistent results with seemingly similar methodologies. In an effort to streamline the utility of this model for the broader research community, we partnered with The Michael J. Fox Foundation for Parkinson's Research (MJFF) to reduce variability and provide a validated tool to be distributed to the research community. Herein, the intent was to assess numerous vector batches in two distinct research groups, sharing tissue and cross-analyzing data to ensure rigor and reproducibility of this model. We assessed several variables, including AAV serotype, genome structure, and promoter construct. Our final selection consisted of a self-complementary AAV genome, carrying the hybrid chicken beta-actin promoter, packaged into AAV5. The resultant construct provides for dose-dependent nigrostriatal degeneration and associated indices such as neuroinflammation and behavioral impairments. Moreover, as reported by others, we observed significant toxicity using a reporter fluorophore as a control; instead, we generated and validated a null vector as the accompanying control. These vectors and plasmid genomes are available for distribution via MJFF.

## Refining the virally-delivered alpha-synuclein model of Parkinson's disease

The protein alpha-synuclein (α-syn) is a vetted participant in the Parkinson's disease (PD) process. However, the exact role of this protein in disease is largely unknown. To better study how this protein may be involved in PD etiology, scientists rely upon animal models that recapitulate features and consequences of pathological α-syn, similar to that seen in human PD. Such features include the formation of protein clumps, inflammation, and ultimately neuronal death and motor dysfunction. One such model has been the approach to utilize gene therapy to increase production of the human form of α-syn in relevant brain areas of rodents. This is achieved by generating gene delivery vehicles in the form of viruses engineered to produce the protein following surgical delivery of the virus. The most common virus utilized for this purpose has been Adeno-associated virus (AAV). However, despite the high utility of this model of PD, reports in literature have been relatively varying in relation to the extent on neuronal death and dysfunction, which has created a barrier for the ubiquitous use of this model in PD research. One reason for this variability stems from the fact that AAV exists in many different forms (aka serotypes) that differ in how effectively they infect neurons and thus levels of α-syn production. Moreover, variations in production methodologies and other esoteric aspects of AAV engineering can also result in viruses with differing efficacy. In this work we partnered with The Michael J. Fox Foundation for Parkinson's Research (MJFF) to reduce variability and provide a validated tool for the research community. In doing so, we identified the ideal version and dose of AAV that yields reliable i) PD-like neuronal death, and ii) Motor dysfunction. These viruses are now made available via the MJFF for the broader research community.

## Introduction

The protein alpha-synuclein (α-syn) is by far the most vetted component of Parkinson's disease (PD) etiopathology. Mutated forms and genetic multiplications of the protein are found in familial forms of the disease.^1–3^ Aggregated α-syn is also a key component of the histopathological landmark of disease–Lewy pathology.^4^ Therefore, this protein is postulated to also play a role in idiopathic disease. The exact role of α-syn in disease remains enigmatic, where both direct toxic gain-of-function from forming harmful multimers and loss-of-function caused by sequestration into these multimeric forms being proposed.^5–7^ Part of the difficulty in understanding the role of α-syn in disease may relate to our limited understanding of the endogenous function of the protein in neurons. α-syn binds to membranes of a specific curvature (e.g., synaptic vesicles) and interacts with various aspects of the synaptic machinery. As such, one of the most well-studied aspects of α-syn is its role in neurotransmission (reviewed in^8^). However, an increasing body of research also suggests a role for α-syn in the nucleus, where the protein binds DNA, possibly due to its inherent curvature, and may be involved in processes such as epigenetic regulation and DNA repair.^9–11^ Regardless, if one surveys the synuclein literature, the protein seemingly interacts with most cellular organelles,^12^ and dissecting potential epiphenomena from real function remains a difficult task. In addition to such cell-autonomous factors, there are other significant aspects of PD that are less amenable to in vitro experimentation, including neuronal dysfunction within the context of a complex circuit, selective vulnerability, spread of pathology, and progressive degeneration that leads to motor and non-motor dysfunction, that would greatly benefit from the use of an animal model with a high degree of face validity.

To aid in the understanding of the role of functional and aggregated α-syn, numerous animal models based on the protein have been created. Initial models were standard transgenic models overexpressing mutant forms of the protein under ubiquitous neuronal promoters.^13^ Additionally, the administration of preformed α-syn aggregates (aka pre-formed fibrils) has evolved to generate a heavily utilized model of α-syn pathology.^14,15^ However, although this model exhibits key pathological features of PD such as post-translational modification of endogenous α-syn and progressive nigrostriatal degeneration, a chief focus of this model is that of extracellular α-syn and its putative spread from cell to cell. Furthermore, the pathology occurs in endogenous rodent α-syn which prevents barriers for testing therapeutic candidates directed towards the human form of the protein. Newer refinements of this model have included the combination with genetic overexpression systems, resulting in a much more rapid degeneration phenotype.^16–19^

Finally, virally-mediated overexpression of human α-syn in rodents and primates has evolved as a useful tool to study cell-autonomous effects of α-syn pathology and aggregation, nigrostriatal integrity and vulnerability, as well as a means to study the function of the protein in a disease-free system by using very low levels of overexpression.^20–25^ Although lentiviruses (LV) were used in the early days of this model's implementation, the majority of current studies are based on adeno-associated viruses (AAV) due to their superior stability, infectivity, and ability to diffuse within the tissue compared to LV.^26,27^ However, despite the value many associate with this model, others have been hesitant or unsuccessful in implementing it due to high variability or an absence of nigrostriatal denervation.^28,29^

One key reason for the disparate results in the use of this model stems from the fact that AAV is a complex system incorporating various components, leading to heterogeneity in systems. For instance, different AAV capsid serotypes infect different neuronal populations with varying efficacy,^30–36^ a plethora of genetic constructs, differing in promoters and non-coding elements, have been used to overexpress α-syn. Finally, different production methodologies, although yielding the same number of genome copies, can differ in the bioactivity of the produced vector (e.g., partially packaged genomes can still be detected with standard titering methods).^37,38^ Moreover, older titering methods such as dot-blot and qPCR have been notoriously varying in their results.^39^ Together, these variables have created a situation where this otherwise powerful and complementary model has led to instances of failures and skepticism in the PD research community.

In an effort to re-envision this model, we partnered with The Michael J. Fox Foundation for Parkinson's Research (MJFF) to design, develop, and validate a new AAV-based model expressing wild-type human α-syn, generating a valuable research resource for the scientific community. In doing so, we empirically assessed several key aspects of this model. First, all work was done in two different laboratories (Sweden and the United States), with overlapping quality control (QC) and with tissue shared between groups in order to enhance rigor, and to ensure that data was reproducible when two groups with different injection parameters performed the experimentation. Moreover, we assessed numerous AAV capsids paired with key genetic elements as an additional variable. We performed parallel experiments in order to arrive at doses that would yield mild or moderate degeneration. Finally, we also assessed several control vectors to identify the transgene that provides the least toxicity at high doses. It is now common knowledge that the use of fluorophores as a control in vulnerable cells such as A9 neurons can result in significant toxicity and therefore confound any experimentation with a target transgene.^16,21,40–42^

**Figure 1.**
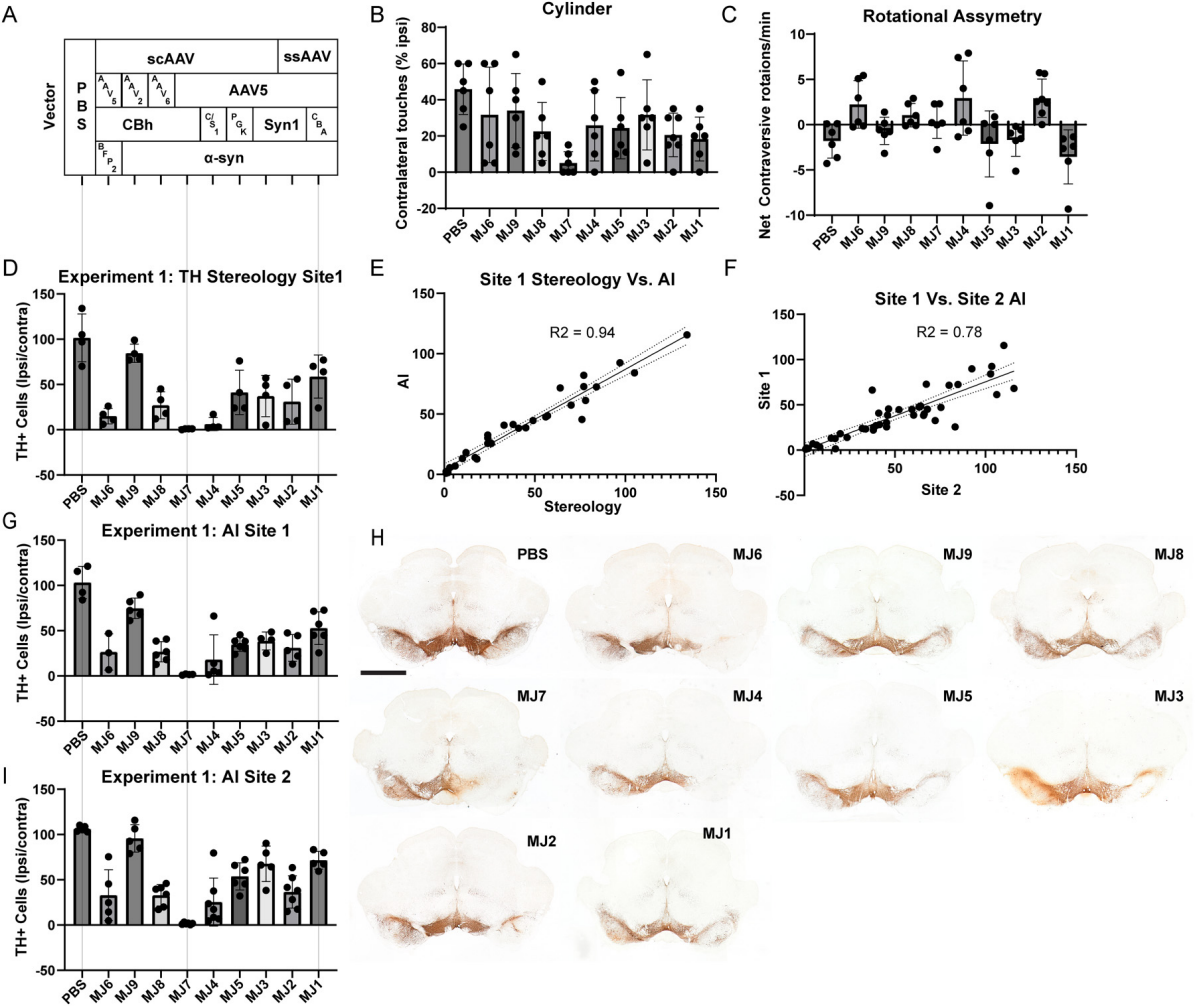
Experiment 1: construct optimization. A variety of AAVs, as outlined in Table 2, were injected into the SNc, and 4 weeks later, animals were tested for motor impairments. **A.** Schematic representation of the various genomes, aligned with all the graphs below (as indicated by vertical grey lines). scAAV5-CBh-α-syn differed from PBS-injected control animals in forepaw cylinder testing (**B**) but not with amphetamine-induced rotations (**C**). Following sacrifice, we performed TH immunohistochemistry to assess nigrostriatal integrity. Although our chief focus was on utilizing AI as a means to enumerate cells, we also performed standard stereology on a subset of animals (**D**). We thereafter utilized two distinct AI algorithms to enumerate TH IR cells on the same sections at both Site 1 (**G**) and Site 2 (**I**). Regression analyses show a high concordance between stereology and AI (**E;** R^2^ = 0.94) and between the different AI algorithms (**F**; R^2^ = 0.78). **H**. Representative midbrain images of TH IR from all injection groups. Scalebar in H = 2.0 mm and applies to all images.

We tested three distinct serotypes: AAV2, as it was one of the original capsids used for this model, AAV5, which reportedly also has a high preference for nigral dopamine neurons,^34^ and AAV6 as it has been used by several groups for α-syn overexpression.^20^ Of note, there are other capsid serotypes that exhibit similar levels of transduction efficacy that were not tested herein. We also compared single-stranded (ss) with double-stranded (self-complementary; sc) AAV, the latter effectively circumventing the rate-limiting second-strand synthesis in vivo, increasing the efficiency and kinetics of the transduction.^43^ In terms of promoters, we assessed the ubiquitous CAG (chicken beta-actin-cytomegalovirus hybrid) promoter (pCBh for scAAV and pCAG for ssAAV), the PGK (phosphoglycerate kinase) promoter, CMV (cytomegalovirus) promoter, and the neuronal synapsin1 promoter. In initial studies, we used mTagBFP2 as our control, but in later studies, we used an AAV genome lacking a protein-coding open reading frame (null virus) as our control. Here we performed our validation in 4 distinct experiments (Table 1), performed in tandem in two independent laboratories, and with materials shared between groups. In Experiment 1 we performed construct optimization, assessing each permutation as indicated (Figure 1, Table 2) with a 4-week survival. Our main outcome measure was nigrostriatal degeneration; moreover, in this Experiment we also validated our artificial intelligence (AI)-based quantitation of neurons. In Experiment 2, we performed a larger-scale assessment of the selected capsid/genome combination, assessing motor impairment and nigrostriatal integrity after a 6-week survival. In Experiment 3, we assessed the final production batch, focusing on identifying doses with distinct characteristics across multiple survival times, as well as evaluating motor function. Finally, in Experiment 4, we assessed the nigrostriatal integrity following the delivery of increasing doses of our null virus control.

**Table 1.** General experimental outline.

Vector batch	Reported qPCR titer (vg/ml)	DdPCR titer (vg/ml)	Dose
Construct optimization	Variable	7.9 × 10^12^	300%
Pilot batch α-syn + tagBFP	2.0 × 10^12^	3.7 × 10^12^	150%
Production batch α-syn + tagBFP	2.0 × 10^12^	2.5 × 10^12^	100%
Production batch 66%	1.3 × 10^12^	1.7 × 10^12^	66%
Production batch 33%	6.7 × 10^12^	8.0 × 10^11^	33%
Null vector [production titer]	6.0 × 10^12^	3.8 × 10^12^	150%
Null vector [stock titer]	3.0 × 10^12^	2.3 × 10^12^	100%

All experiments, regardless of site, utilized the same general injection paradigm. Exp. 1 utilized a 4-week survival, Exps. 2 and 4 utilized a 6-week survival time, and Exp. 3 utilized both 6- and 12-weeks survival. In all Experiments, TH neuron survival and motor function were assessed. qPCR vector titers reported by GeneDetect were validated using ddPCR.

**Table 2.** Construct optimization vector permutations.

Name	Genome structure	Capsid	Promoter	Transgene	N
PBS	N/A	N/A	N/A	N/A	5
MJ1	SS	2/5	CBA	α-syn	5
MJ2	SS	2/5	Synapsin1	α-syn	7
MJ3	SC	2/5	Synapsin1	α-syn	5
MJ4	SC	2/5	CMV	α-syn	7
MJ5	SC	2/5	PGK	α-syn	6
MJ6	SC	2/5	CBh	mTagBFP2	2
MJ7	SC	2/5	CBh	α-syn	6
MJ8	SC	2/6	CBh	α-syn	6
MJ9	SC	2/2	CBh	α-syn	5

Abbreviations: SS = single stranded, SC-self-complementary, CBA-chicken beta-actin, CMV-cytomegalovirus, PGK- phosphoglycerate kinase, CBh-Truncated CBA promoter.

### Materials and methods

To enhance the rigor of our model validation, all work took place at two independent sites: Site 1: Barrow Neurological Institute, USA; Site 2: Lund University, Sweden. To complete the experimentation, we performed 4 distinct Experiments (Table 1), where tissue was shared and/or data was analyzed across both sites.

#### Recombinant genomes

The final recombinant α-syn (pscAAV-CBh-aSyn-WPRE3-enSV40pA; Addgene:194244), tagBFP2 (pscAAV-CBh-tagBFP2-WPRE3-enSV40pA; Addgene:194246), and Null (pscAAV-CBh-Null-WPRE3-enSV40pA; Addgene:194245) genomes were deposited into Addgene for dissemination (WWW.Addgene.Org).

#### Vector production

Self-complementary adeno-associated virus serotype 5 (scAAV5) vectors were supplied by GeneDetect Ltd (Auckland, New Zealand), produced using standard methodology.^44–46^ Briefly, plasmids encoding recombinant genomes, capsids, and helper functions were transfected into HEK293 cells, followed by harvest 68–76 h later. Viral particles were purified using a discontinuous iodixanol gradient and dialyzed using a 100 kDa molecular weight cut-off Amicon concentrator using modified PBS supplemented with 0.001% Pluronic acid. The final product was stored in PBS-MK with 0.001% Pluronic acid. The viral vectors from Experiment 4 are available through the University of Iowa Viral Vector Core (scAAV2/5 Hu WT α-syn, MJFF-GD1006; scAAV2/5 Empty/Null, MJFF-GD1007).

#### Vector QC

GeneDetect: Vector titers were determined using the Applied Biosystems QuantStudio 12 K Flex Real-Time PCR System using primers designed to amplify a 123 bp amplicon from within the CBh promoter region (Fwd (5′ to 3′): CCCACTTGGCAGTACATCAA and Rev (5′ to 3′): GCCAAGTAGGAAAGTCCCATAA). For quantitative PCR, triplicate vector samples were treated with DNase I (New England Biolabs) for 30 min at 37°C to remove unpackaged DNA. PCR reactions were then performed using Power SYBR Green Master Mix (Applied Biosystems). A plasmid standard curve was used to calculate the genomic titer (vector genomes (vg)/mL). Given the often-reported variability in titering methodology,^39^ especially qPCR, all batches were subject to droplet digital PCR (ddPCR) titering using our previously developed methods^45^ (Table 1). ddPCR data was thereafter used to normalize all vector batches in reference to the final production batch which was 2.5 × 10^12^ vg/ml. This dose is referred to as 100% throughout the manuscript, and all other titers reported throughout are in reference to this titer. All titers can be found in Table 1.

#### Animals

Site 1: Young adult (∼3-month-old) Sprague-Dawley (Envigo) rats were utilized, and all procedures were in accordance with the Barrow Neurological Institute Institutional Animal Care and Use Committee. Site 2: Three-month-old female SD rats (Taconic Biosciences) were used. The procedures were approved by the Ethical Committee to use Laboratory Animals in the Lund-Malmö region. Rats were housed two per cage, maintained on a controlled 12-h light cycle and temperature (22°C) environment. Food and water were available *ad libitum*.

#### Vector injections

Stereotaxic delivery of AAVs was performed similarly across sites, using siliconized glass capillary needles fitted to a Hamilton Gas Tight syringe (Hamilton Gas Tight syringe 80,000, 26 s/2” needle [Hamilton]).^47^ The surgical procedure was performed under isoflurane anesthesia (1.7–2.0%) with the rats’ skulls secured in a stereotaxic frame. Surgical coordinates were anterior/posterior −5.3 medial/lateral + 1.8 mm, dorsal/ventral (from dura) −7.2 mm. During the injection, the capillary was lowered to the injection sites, and 3 µl of vector was injected at a rate of 0.4 µl/min. Following vector delivery, the needle remained in place for 5 min before retracting.

#### Behavioral testing

##### Cylinder test

The cylinder test was used to assess forelimb asymmetry in exploratory behavior.^48,49^ The rats were placed in a glass cylinder (21 cm in diameter and 34 cm in height) and recorded for 5 min. Afterwards, the number of right and left forepaw touches were counted by a blinded researcher. Data is presented as contralateral touches as a percentage of total touches.

#### Corridor test^50^

The animals were food-restricted for 3 days (90% free-feeding body weight), placed into the corridor (240 cm long×7 cm wide×23 cm deep) with food pellets and recorded for 5 min. A retrieval is counted when the animal uses its nose to explore the food basket. Afterwards, the number of right and left retrievals was counted by a blinded researcher. Data is presented as contralateral food retrievals as a percentage of total retrievals.

#### Drug-induced rotational asymmetry^51^

Rats were injected i.p. with 2.5 mg/kg d-amphetamine (Apoteksbolaget) and placed in automated rotameter bowls. Rotations were recorded for 90 min. The data are presented as net ipsiversive rotations per minute.

#### Euthanasia

Site 1: At the assigned times following vector delivery, animals were sacrificed in accordance with the American Veterinary Medical Association and as approved by the Barrow Neurological Institute Institutional Animal Care and Use Committee (IACUC). Rats were deeply anesthetized with 60 mg/kg pentobarbital (i.p.) and thereafter transcardially perfused with Tyrode's solution followed by ice-cold 4% paraformaldehyde. Following removal, the brains were placed in 4% paraformaldehyde for 24 h and thereafter cryoprotected in 30% sucrose until saturated. Brains were sectioned coronally using a sliding stage microtome into 6 serial 40 μm sections and stored in cryoprotectant at −20°C until use.

Site 2: Animals were first deeply anesthetized with sodium pentobarbital overdose (Apoteksbolaget, Sweden), then transcardially perfused with 50 mL of physiological saline solution followed by 100 mL of 4% paraformaldehyde (PFA). Brain tissue was extracted and postfixed for 24 h at 4°C and subsequently stored in 25% sucrose in 0.1 M phosphate buffer (pH 7.4). The brains were sectioned in a microtome at 40 μm thickness into 6 series. The sections were kept in cryoprotectant at −20°C until staining.

#### Immunostaining of brain sections

Site 1: All immunohistochemistry (IHC) and immunofluorescence was performed as previously described^52^ and washed with TBST (tris buffer saline with triton X100, 0.25%) between each step. Briefly, sections were quenched with 0.3% H_2_O_2_ (IHC) followed by blocking in appropriate serum. Sections were thereafter incubated in the primary antibody (Table 3) overnight at room temperature (RT) followed by the appropriate secondary antibody for 2 h in RT. Sections were incubated with an avidin-biotin complex as per manufacturer instructions (Vector ABC kit) and developed using 3,3′ diaminobenzidine and 0.03% hydrogen peroxide in tris buffer. Sections were mounted on subbed slides, dehydrated using increasing concentrations of ethanol followed by xylene and coverslipped using Cytoseal™ (Fisher Scientific).

**Table 3.** Antibodies utilized.

Primary antibody				Secondary antibody		
Antigen	Host/Class	Cat#	Dilution	Host/Class	Cat#	Dilution
α-syn (sites 1 and 2)	Mouse monoclonal	Invitrogen AHB0261	1:2000	Horse Anti-Mouse IgG Antibody, rat adsorbed (H + L), Biotinylated	Vector Laboratories BA-2001-.5	1:500
				Goat anti-Mouse IgG1 Cross-Adsorbed Secondary Antibody, Alexa Fluor™ 568	Invitrogen A-21124	1:500
TH (sites 1 and 2)	Rabbit polyclonal	Millipore #AB152	1:4000	Goat Anti-Rabbit IgG Antibody (H + L), Biotinylated	Vector Laboratories BA-1000-1.5	1:500
Iba1 (site 1)	Rabbit polyclonal	Fujifilm Wako 019-19741	1:2000	Goat Anti-Rabbit IgG Antibody (H + L), Biotinylated	Vector Laboratories BA-1000-1.5	1:500
				Goat anti-Rabbit IgG (H + L) Cross-Adsorbed Secondary Antibody, Alexa Fluor™ 647	Invitrogen A-21244	1:500
GFAP (site 1)	Mouse monoclonal	Abcam ab279289	1:2000	Horse Anti-Mouse IgG Antibody, rat adsorbed (H + L), Biotinylated	Vector Laboratories BA-2001-.5	1:500
GFAP (site 1)	Chicken monoclonal	Abcam ab323239	1:2000	Goat anti-Chicken IgY (H + L) Secondary Antibody, Alexa Fluor™ 488	Invitrogen A-11039	1:500
HuC/ HuD (site 1)	Mouse monoclonal	Invitrogen A-21271	1:2000	Goat anti-Mouse IgG2b Cross-Adsorbed Secondary Antibody, Alexa Fluor™ 488	Invitrogen A-21141	1:500
OLIG2 (site 1)	Rabbit monoclonal	Invitrogen MA5-42372	1:2000	Goat anti-Rabbit IgG (H + L) Cross-Adsorbed Secondary Antibody, Alexa Fluor™ 647	Invitrogen A-21244	1:500
α-syn S129 (site 1)	Rabbit monoclonal	Abcam ab51253	1:1250	Goat Anti-Rabbit IgG Antibody, biotin-SP conjugate	Millipore Sigma AP132B	1:500
aSyn (site 2)	Mouse monoclonal	Santa Cruz SC12767	1:2000	Horse Anti-Mouse IgG Antibody, rat adsorbed (H + L), Biotinylated	Vector Laboratories BA-2001-.5	1:500
mTag BFP2 (site 2)	Mouse monoclonal	Invitrogen MA5-15257	1:2000	Horse Anti-Mouse IgG Antibody, rat adsorbed (H + L), Biotinylated	Vector Laboratories BA-2001-.5	1:500

Site 2: Sections were removed from antifreeze solution and washed in PBS three times while shaking. Sections were then placed in 1 ml quenching solution (10% methanol, 3% H2O2 in PBS; pH = 7.4) for 1 h. After quenching, the sections were washed three times in PBS. Sections were then incubated in blocking solution (5% serum of secondary antibody species + 0.25% Triton in PBS) for 1 h at room temperature. Following blocking, the samples were incubated with primary antibody (Table 3) diluted in blocking solution at room temperature overnight or at 4 °C for 48 h. After incubation with the primary antibody, the sections were washed three times in PBS. Thereafter, they were incubated in blocking solution for 15 min, followed by incubation with secondary antibody diluted in blocking solution for 2 h at room temperature. The ABC Complex (Vector Labs elite, PK-6100) was prepared at least 30 min before its use. Sections were washed in PBS three times and then incubated in ABC complex solution for 1 h. Sections were washed in PBS three times and then incubated with DAB solution using the DAB Peroxidase Substrate Kit (#SK-4100, Vector Laboratories) according to the manufacturer's protocol. The sections were washed three times in PBS and then mounted onto coated slides and left to air dry. The slides were then dehydrated and subsequently covered using DPX (Sigma Aldrich).

#### Imaging

Site 1: Slides for artificial intelligence (AI)-based quantitation of TH + cells (full series) were imaged on a ZEISS Axioscan (ZEISS Group; Oberkochen, Germany). Each scan consisted of a series of image tiles acquired across the X-Y plane of the SN. In addition, each tile consisted of a stack of images acquired with the 20X objective across 13 µm on the Z-axis with a 0.8 µM step size. Finally, acquired photomicrographs were processed and stitched together by the ZEN^®^ advanced software, producing a single high-resolution digital image per each scan. Image files were uploaded to AIforia (AIforia technologies, Finland) for subsequent enumeration of TH + cells^53^ (Supplemental Figure 1). Other brightfield images were acquired on a Nikon Eclipse Ni microscope. Fluorescent images were acquired using a Nikon A1R HD25 confocal microscope equipped with a 60× oil-immersion objective (NA 1.4) and Z-stack images were collected at 0.75 μm intervals across the tissue section. Images were processed using NIS-Elements software (Nikon Instruments Inc.). Representative images were selected from subjects that displayed mean values in terms of indicated outcome measures.

Site 2: 2X image overview and 20X scans were acquired with an Olympus BX61VS microscope, using the Olympus VS-ASW 2.9 software, subsequently .vsi files were uploaded to the AIforia cloud (AIforia technologies, Finland). In that platform, an AI model using deep CNN algorithms with supervised learning was deployed for TH + cells count in the SNpc. For the model, only a layer type object with “very complex” complexity was used to detect the mentioned cells.

#### AI-based enumeration of Th immunoreactive cells within the Sn

Sections were uploaded to an image analysis platform developed by AIforia (AIforia Technologies; Helsinki, Finland), which uses individualized AI deep convolutional neural network (CNN) learning to facilitate quantitative histological image analysis.^54^ Briefly, high-resolution brightfield photomicrographs acquired using ZEISS Axioscan were imported into AIforia software. The SN was outlined using a contour tool in AIforia. A custom-developed deep learning process was used to train a convolutional neural network to enumerate the total number of TH IR cells (Supplemental Figure 1).

#### Densitometric analysis of GFAP and Iba1 immunoreactivity

High-resolution brightfield photomicrographs were acquired using the ZEISS Axioscan.Z1 system. Quantification of staining intensity was performed using FIJI/ImageJ software (National Institutes of Health). Prior to analysis, all images were subjected to 8-bit grayscale inversion to optimize pixel intensity mapping, followed by background subtraction using a rolling ball radius of 50 pixels to minimize non-specific signal. Quantitative measurements, including area, mean gray value, minimum and maximum gray values, and threshold limits, were configured and uniformly applied across all images to ensure consistency in signal-to-noise ratios. Regions of interest (ROIs) were manually delineated with reference to the Paxinos and Watson rat brain atlas (from bregma: +2.52 to −1.56 mm and −4.56 to −6.48 mm),^55^ allowing for precise anatomical localization. Once ROIs were defined, densitometric analysis was performed using the Measure function. The resulting data, particularly the mean gray value, were used as the primary metric for quantifying staining intensity and were exported for statistical analysis. Data are reported as arbitrary units as a ratio between hemispheres.

#### RNAscope ISH of for validation of transduction

To assess transduction of the control null vector in Experiment 4, we performed *in situ* hybridization (ISH) with a probe designed against the WPRE element. ISH was carried out using the Advanced Cell Diagnostic RNAscope^®^2.5 HD detection Kit -Brown (ACD, Cat#322310) and probe WPRE-O7-C1 (ACD Cat# 1147131-C1). Protocol was carried out as suggested by the manufacturer's instructions with a few modifications for 40uM free-floating frozen sections as described previously.^56^ Briefly, the cryoprotectant was removed by six 5-min TBS washes. Sections were then incubated with peroxide treatment for 45 min at room temperature and washed 3 × 5 min. Sections were mounted onto Superfrost Plus + slides and air-dried, then washed×3 in H_2_O to remove salts and air-dried overnight at RT. The following day, sections were incubated in 99–100°C target retrieval solution for 10 min followed by 4×wash in H_2_O and 8× 100% ethanol, and allowed to air-dry before drawing a hydrophobic barrier. Next, protease (protease III) treatment, probe hybridization, signal amplification and signal detection steps were carried out exactly as described per the manufacturer's protocol. After the final rinse, sections were allowed to air-dry overnight at RT. Slides were dehydrated and coverslipped as described above.

#### Statistics

Our only exclusion criterion (absence of transduction) was established *a priori.* All data were collected by experimenters blinded to the experimental conditions. In Exp. 1, a one-way analysis of variance (ANOVA) was used, followed by Tukey's HSD for multiple comparisons. In Exp. 2, data were analyzed using an unpaired, two-tailed t-test. In Exp. 3 a two-way ANOVA test was used to detect statistical significance between all groups with vector (α-syn/BFP2) and time (6, 12 weeks) as the independent variables. To further define any relationship across, within, and between variables, Šídák's multiple comparisons post hoc test was used, where a main or interaction factor significance was found. For all analyses, alpha level of 0.05 was considered statistically significant. Finally, simple regression analyses were performed to compare and validate results as noted. All statistical analyses were conducted using Prism (GraphPad) and graphs are presented as the mean with SD error bars.

## Results

### Experiment 1- construct optimization

In our initial experiment, we performed a head-to-head comparison of vectors with the following variables (Tables 1,2): 1) scAAV versus ssAAV. 2) Serotypes 2, 5, and 6. 3) Promoters- truncated CBA (CBh), CMV, PGK, synapsin1, and full-length CBA. 4) AAV5-BFP as a control for transduction (Figure 1A). Vector injections and behavioral analyses were performed at Site 2, and the survival time was four weeks. TH stereology was done at Site 1, and AI-based enumeration of TH + neurons at both sites.

#### Titer determination

All vectors were titered using ddPCR, and for Experiment 1 vector titers were normalized to 7.9 × 10^12^ vg/ml based on ddPCR results (300%).

#### Behavioral analyses

Animals were tested for forelimb asymmetry and amphetamine-induced rotations. Results are summarized in Table 4. Briefly, a 1-way ANOVA (F _(9, 51)_ = 2.45; p = 0.02) revealed that in the cylinder test (Figure 1B), only the scAAV5-CBh-α-syn (MJ7) injected group exhibited an impairment as compared to the PBS injected control (p < 0.005) with no difference seen between vectors. No significant impairments were observed in the amphetamine-induced rotational test (Figure 1C).

**Table 4.** Experiment 1 results.

% contralateral				Rotations/min	
Vector	Stereology Site 1	AI Site 1	AI Site 2	Cylinder Site 2	Rotations Site 2
PBS	101.5*	103.3*	106.1**	45.8 #	-1.8 ##
MJ1	58.8	52.9	71.6	18.3	-3.6
MJ2	31.0	31.0	36.5	20.6	2.9
MJ3	37.3	38.7	67.7	31.7	-1.7
MJ4	6.3	18.1	25.4	25.8	2.9
MJ5	41.3	34.8	53.8	24.3	-2.1
MJ6	15.0	26.5	33.0	31.7	2.2
MJ7	0.8	1.6	1.6	5.0	0.4
MJ8	27.0	26.7	32.9	22.5	1.1
MJ9	84.5	74.7	95.7	34.1	-0.68

*P < 0.005 versus all groups except MJ1and MJ9. **p < 0.05 versus all groups except MJ1 and MJ9. #p < 0.005 vs MJ7. ##p < 0.05 vs MJ2.

#### Histological analyses

Quantitation of TH + cells in the SNc is summarized (as a ratio of ipsilateral counts/contralateral counts) in Table 4. Briefly, all vector groups differed from the PBS control, including the BFP2 control group, except for MJ1 and MJ9. Nevertheless, in all analyses, the scAAV5-CBh-α-syn group exhibited near-complete nigral denervation.

**Figure 2.**
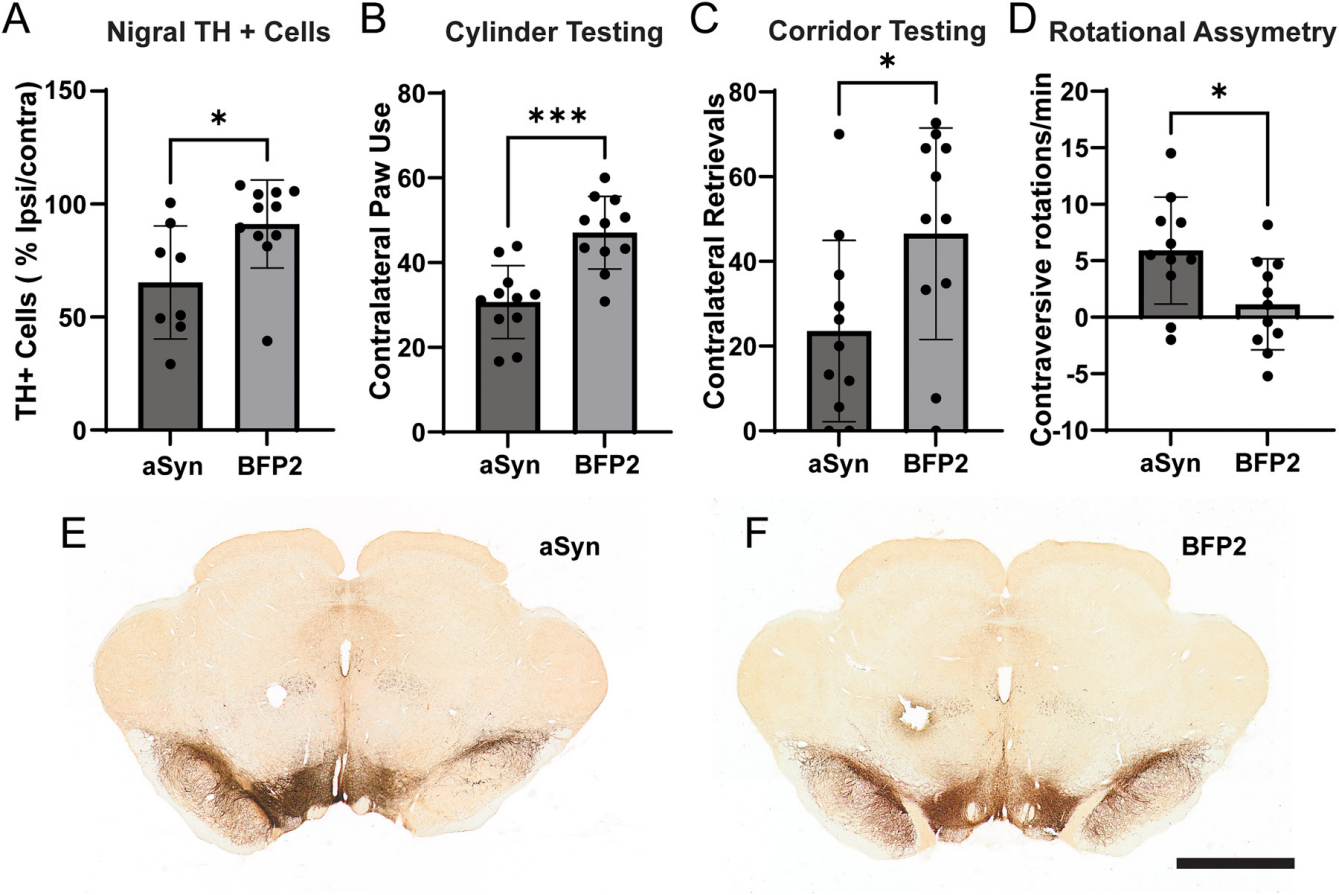
Experiment 2: assessment of lead vector candidate. A pilot production batch of scAAV5-CBh-α-syn or BFP2 was produced and we performed a more in-depth analysis at a lower titer than in Exp.1 with a 6-week survival. α-syn expressing animals showed a significant reduction in midbrain TH + neurons (**A**), a significant reduction in contralateral forepaw use in the cylinder test (**B**), a significant deficiency in the corridor test (**C**), and significant ipsiversive turning (**D**). Representative images of midbrain TH immunoreactivity in α-syn treated (**E**) and BFP2 (**F**) treated animals. * p < 0.05; *** p < 0.001 (unpaired t-test). Scalebar in F = 2.0 mm and applies to both images.

Moreover, we performed several regression analyses to validate the AI algorithm and multi-site approach. Validating the AI algorithm by comparing Site 1 stereology results (Figure 1D) with AI-based enumeration (Figure 1G) yielded a highly significant correlation between the two approaches (Figure 1E; R^2^ = 0.94; P < 0.0001). Similarly, we also compared the AI results between sites 1 (Fig. 1D) and 2 (Fig. 1I), again displaying/revealing a significant correlation in the estimated TH + neuron population (Fig. 1F; R^2^ = 0.78; P < 0.0001). Figure 1H displays representative nigral TH immunoreactivity for all groups as described in Table 2 and Figure 1I.

### Experiment 2 – pilot batch assessment

Based on the results from Exp. 1, we decided to utilize scAAV5-CBh-α-syn (n = 11) as our lead vector (and scAAV5-CBh-tagBFP2 as control; n = 11) for which a larger pilot batch was generated. Given the significant toxicity seen with our control vector in Exp. 1, titers in this second experiment were reduced to 3.1 × 10^12^ vg/ml (150%). Animals received the same unilateral injection paradigm as in Exp. 1 but with a 6-week survival period. Moreover, animals were assessed for motor dysfunction (cylinder test, rotational asymmetry) and lateralized sensorimotor neglect using the corridor test.

#### Histology

The extent of nigral degeneration in Exp.2 was analyzed using the AI algorithm developed by Site 2 (Figure 2A). α-syn overexpression was associated with significant loss of midbrain TH + neurons (Figure 2E; 34.7% loss) as compared to control (Figure 2F; 8.8% loss; p < 0.05).

#### Behavioral analyses

Animals receiving the α-syn vector exhibit behavioral impairments in all tests performed at 6 weeks. In the cylinder test (Figure 2B) α-syn animals exhibited 30.7% contralateral paw use versus 47.1% for controls (p < 0.0005). In the corridor test (Figure 2C), α-syn expressing animals exhibited fewer contralateral retrievals as compared to controls (23.6 versus 46.5, respectively; p < 0.05). Finally, in the amphetamine-induced rotational test (Figure 2D) α-syn animals exhibited 5.9 ipsiversive rotations/minute, and controls 1.1 ipsiversive rotations/minute (p < 0.05).

### Experiment 3 – production batch assessment

Following the positive results obtained in Exp. 2, we obtained aliquots of the final production batch for validation. Injections were performed at both sites using the same general parameters. Site 1 injected 3 different doses (33%, 66%, and 100%; 6-week survival) and tissue was sent to Site 2 for quantitation of TH + midbrain neurons using AI. Site 2 (12-week survival) injected a single dose (66%) and assessed animals for behavioral impairments at 6 and 12 weeks.

#### Behavioral analyses (Site 2)

**Figure 3.**
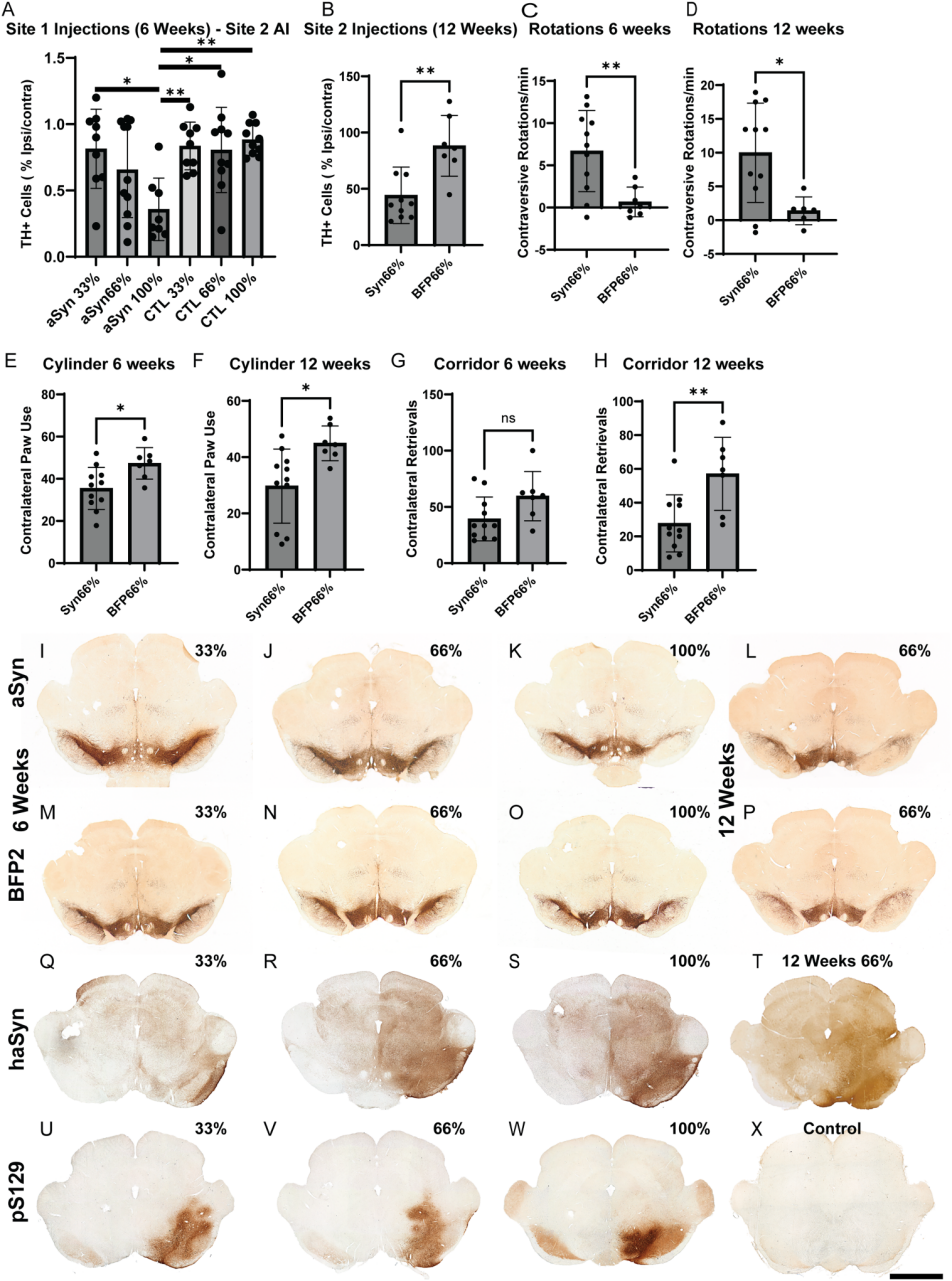
Experiment 3: dose-assessment of lead vector final production batch. A final batch of scAAV5-CBh-α-syn or BFP2 was produced and various doses were assessed over a survival of 6 (33%, 66%, 100%; Site 1) or 12 weeks (66%; Site 2) where behavior was also assessed at 6 and 12 weeks. **A**. Sections from Site 1 were shipped to Site 2 for immunostaining. AI-based enumeration was used to assess loss of nigral TH + neurons. The α-syn 100% group exhibited significantly fewer ipsilateral neurons than α-syn33% and all control groups (1-way ANOVA). **B**. Animals injected with 66% α-syn observed significantly fewer TH + neurons than controls. The 66% cohort also exhibited a significant motor impairment as assessed by amphetamine-induced rotations at 6 (**C**) and 12 (**D**) weeks. Similarly, contralateral forepaw use in the cylinder test was reduced at 6 (**E**) and 12 (**F**) weeks. No impairment was seen in the corridor test at 6 weeks (**G**), but reduced retrievals were seen 12 weeks after vector delivery (**H**). Representative midbrain TH immunoreactivity in animals treated with 33% (**I**), 66% (**J**), 100% (**K**) 6 weeks after injection, and (**L**) 66% 12 weeks after injection. Corresponding nigral TH immunoreactivity 6 weeks after vector delivery of control vector, 33% (**M**), 66% (**N**), 100% (**O**), and 66% 12 weeks after vector delivery (**P**). Representative human α-syn immunoreactivity 6 weeks after vector delivery in the 33% (**Q**), 66% (**R**), and 100% (**S**), and 12 week 66% (**T**) treatment groups. Representative nigral pS129 α-syn immunoreactivity from the 33% (**U**), 66% (**V**), 100% (**W**), and control (**X**) groups. * p < 0.05, ** P < 0.01. Scalebar in X = 2.0 mm and applies to all images.

Amphetamine-induced rotations (Figure 3 C,D): A two-way ANOVA indicated a significant effect of both vector (F _(1, 16)_ = 10.18; p < 0.006) and time (F _(1, 15)_ = 4.639, p < 0.05). α-syn treated animals, as compared to control, exhibited ipsiversive turning at 6 weeks (6.7 rotations/minute versus 0.7 rotations/minute; p < 0.05) and 12 weeks (10.0 rotations/minute versus 1.3 rotations/minute, respectively; p < 0.005)

Cylinder testing (Figure 3 E,F): A two-way ANOVA analysis revealed a significant effect of vector (F _(1, 16)_ = 10.76, p < 0.005) but not time (F _(1, 16)_ = 2.330, p = 0.15), with α-syn overexpression resulting in fewer contralateral touches, compared to control, at 6 weeks (35.4% and 47.3% respectively; p < 0.05) and 12 weeks (29.7% and 44.9% respectively; p < 0.01).

Corridor test (Figure 3 G,H): A two-way (time×vector; F _(1, 16)_ = 0.5035, p = 0.49) ANOVA showed a significant effect of vector (F _(1, 16)_ = 12.55; p < 0.005), but not time (F _(1, 16)_ = 1.201; p = 0.3). There was no difference in contralateral retrievals at 6 weeks (α-syn: 39.3, BFP: 59.5; p = 0.08), but α-syn animals showed impairment in this task at 12 weeks as compared to control (27.7 versus 57.1, respectively; p < 0.01).

#### Histology

##### Nigrostriatal degeneration

All AI-based quantitation was performed at Site 2. Moreover, 2 animals from each group in the 6-week cohort were also assessed using standard stereology (Site 1); there was significant concordance between the data generated at the two sites (R^2^ = 0.98, P = 0.0005).

6-week time point (Figure 3A): Animals receiving the highest α-syn dose (100% dose; Figure 3 K) exhibited a significant loss of TH + midbrain neurons (35.7% remaining) as compared to the lowest dose of α-syn (33% dose; 81.4% remaining; Figure 3I p < 0.05), but not compared to the intermediate group (66% dose; 65.6% remaining; Figure 3J). The 100% dose group also differed from all control groups (33% dose; 83.6% remaining; Figure 3M, 66% dose; 80.6% remaining; Figure 3N, 100% dose; 88.3% remaining; Figure 3O; p < 0.05). No difference was seen between any of the control groups (p = n.s.).

12-week time point (Figure 3B): At the 12-week end point α-syn overexpressing animals exhibited a significant loss of nigrostriatal neurons (44.2% remaining) versus control (88.2% remaining; p < 0.005; Figure 3L,P).

##### Transduction

A qualitative assessment using the human-specific Syn211 antibody six (Figure 3 Q-S) or twelve weeks (Figure 3T) after transduction aligned largely with the dosing parameter, with increased levels of hα-syn immunoreactivity seen between 33% (Figure 3Q), 66% (Figure 3R), and 100% doses (Figure 3S). No appreciable significant difference was observed between 6 and 12 weeks (Figure 3R & 3 T, respectively). Minor differences were observed in staining performed between sites-the likely result of different antibodies used.

##### S129 phosphorylation

While S129 phosphorylation (pS129) is activity dependent^57^ the presence of pS129 is also associated with aggregated α-syn,^58^ and thus an important marker for pathological α-syn. Significant pS129 immunoreactivity was seen in the midbrain of 33% (Figure 3U), 66% (Figure 3 V), and 100% (Figure 3W) treatment groups. No pS129 was observed in the control group (Figure 3X).

##### Tropism

**Figure 4.**
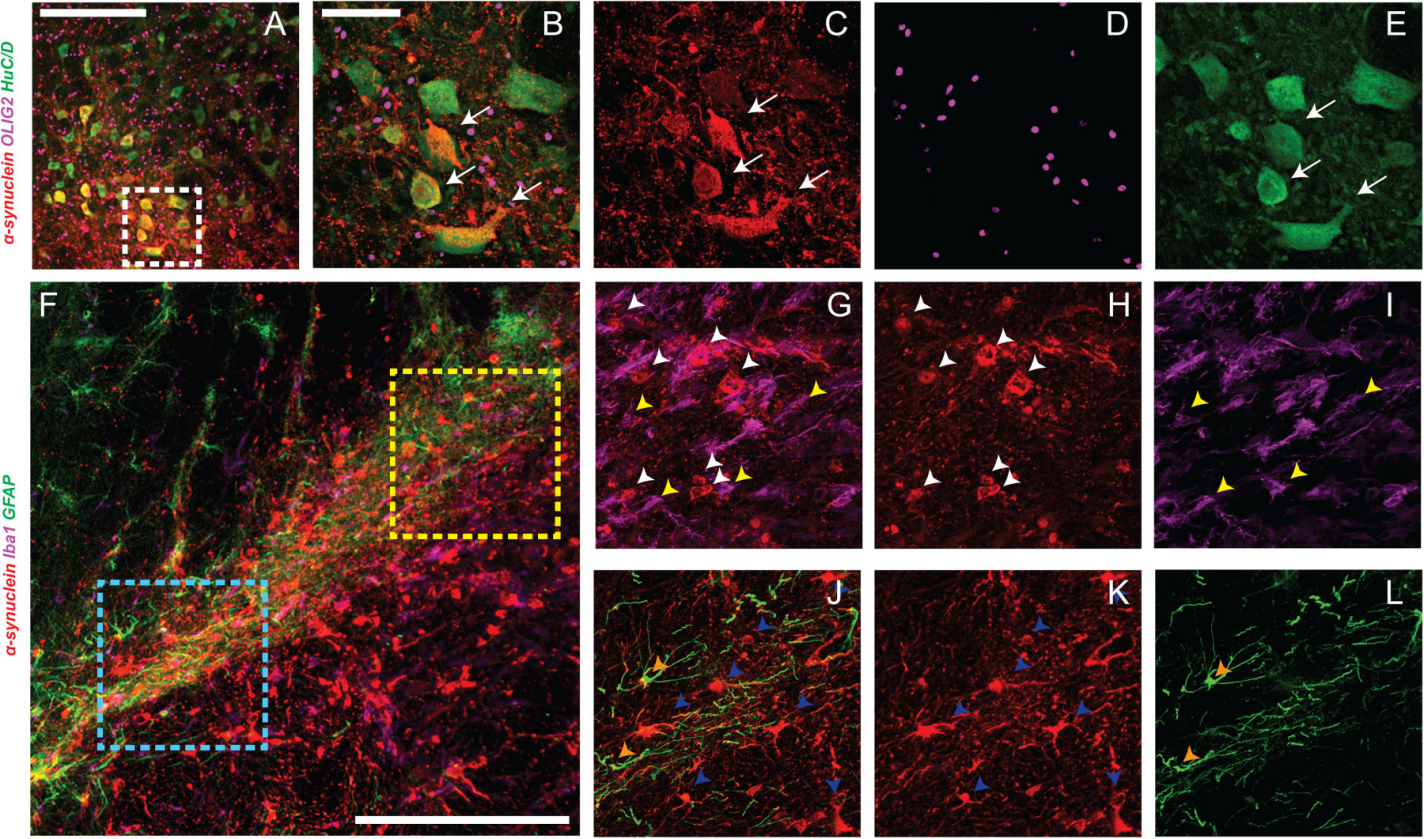
Tropism of AAV-hα-syn transduction. Representative immunofluorescence images illustrating the cellular tropism of scAAV5-CBh-α-syn in rat midbrain. Low magnification images are shown in (A) and (F), with dashed boxes indicating the higher magnification images. The white dashed box in (A) corresponds to the merged image (B) and its single channels, hα-syn (C, red), Olig2 (D, purple), and HuC/D (E, green), showing α-syn expression in HuC/D-positive neurons (white arrows), with no detectable overlap in Olig2-positive oligodendrocytes. The yellow dashed box in (F) corresponds to the merged image (G) and its single channels, hα-syn (H, red) and Iba-1 (I, purple), showing that α-syn-positive cells (white arrowheads) do not colocalize with Iba-1-positive microglia (yellow arrowheads).The blue dashed box in (F) corresponds to the merged image (J) and its single channels, hα-syn (K, red) and GFAP (L, green), showing no colocalization between α-syn-positive cells (blue arrowheads) and GFAP-positive astrocytes (orange arrowheads). Scale bars: 200 µm in (A) and (F) and 50 µm in (B–L).

α-syn pathology in PD is largely associated with neuronal inclusions. In order to validate that transduction emulates this important aspect of etiopathology we also performed a qualitative assessment of capsid tropism of the final scAAV5-CBh construct (66%). As expected,^53,59^ transduction was almost completely neuronal (Figure 4A-E) with little to no overlap of hα-syn expression with microglia (Iba 1; Figure 4F-1), astrocytes (GFAP; Figure 4F, J-L), or oligodendrocytes (Olig2; Figure 4A-E).

### Experiment 4 – null vector assessment

Given that the use of the mTagBFP2 control was not inert, we performed a final experiment to assess a new control vector: a null vector (NV) carrying the exact same genome as the scAAV5-CBh-α-syn vector but the a-Syn open reading frame (ORF) was replaced with a short non-coding reading frame flanked by the human CTE in the 3’ UTR to normalize the length of the two vector genomes. Animals were injected at Site 2 using the same parameters as in earlier experimentation, with animals receiving either 100% dose α-syn, 100% or 150% dose NV followed by sacrifice 6 weeks later.

#### TH

**Figure 5.**
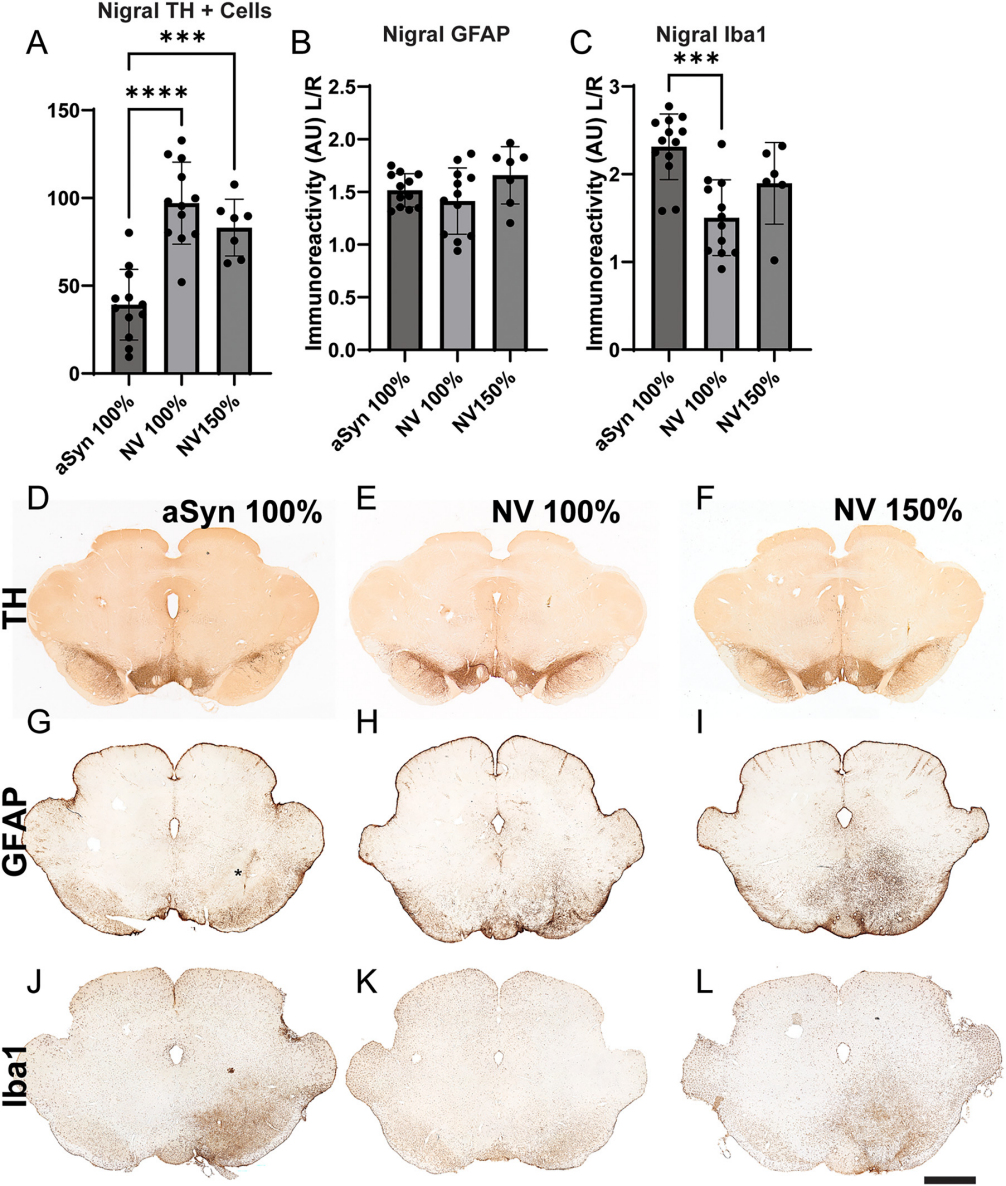
Experiment 4: assessment of null vector. Given that our BFP2 control vector was not completely inert, we performed an additional experiment where we utilized a null vector (empty cassette) in lieu of BFP2 at 100% and 150% and compared this to α-syn (100%). A. AI-based enumeration showed a significant reduction in midbrain TH + neurons with α-syn overexpression compared to both control groups. B. There was no difference in nigral GFAP immunoreactivity between the two groups; however, α-syn overexpression (B) resulted in significantly higher Iba1 immunoreactivity compared to the control. Representative images of midbrain TH immunoreactivity with 100% α-syn (D), 100% NV (E), and 150% NV (F). Representative images of midbrain GFAP immunoreactivity in 100% α-syn (G), 100% NV (H), 150% NV (I) groups. Asterisk in G denotes a rough estimate of the SNc location, an area void of staining. Representative images of Iba1 immunoreactivity in 100% α-syn (J), 100% NV (K), 150% NV (L) groups. **** p < 0.0001, *** p = 0.0001 (1-way ANOVA). Scalebar in L = 2.5 mm and applies to D-L.

Using AI-based enumeration (Site 2, Fig. %A), we observed a significant loss of TH + neurons with α-syn (39.2% remaining; Figure 5D), versus 100% dose NV (97.1% remaining; p < 0.0001; Figure 5E) and 150% dose NV (83.1% remaining; P = 0.0001; Figure 5F). No difference was observed between the 100% dose α-syn groups from Exp. 3 and Exp. 4 (data not shown).

#### Inflammation

In addition to TH neuron survival, we assessed gliosis by densitometric analysis of GFAP and Iba1. No difference in astrogliosis was seen between groups, with a slight increase in the ipsilateral hemisphere (Figure 5B, G-I). It is noteworthy, however, that the α-syn treated group exhibited a void in the staining pattern (centered around the asterisk in Figure 5G), suggesting that perhaps astrogliosis precedes neuronal loss. However, the α-syn treated group exhibited significantly higher Iba1 immunoreactivity (Figure 5C, J-L) as compared to the same dose NV (p < 0.0005). No difference was seen between the two null vector doses.

#### RNAscope

In order to assess transduction between groups, we performed RNAscope *ISH* against the WPRE component of the genome to facilitate the detection of the Null vector (Supplemental Figure 2). Interestingly, densitometric quantitation of the RNAscope signal (Supplemental Figure 2) showed higher *ISH* signal in the α-syn 100% dose group as compared to the 150% dose NV group (P < 0.05). However, a qualitative assessment of the micrographs revealed that for both NV groups, the signal was much more punctate, whereas in the α-syn group, the ISH signal filled the cell to a much greater degree. This suggests that there is a difference in intracellular transport and stability between the two genomes. While both the CTE and WPRE UTR sequences should promote nuclear export, the lack of a complete ORF may still inhibit this process, leaving most of the transcripts in the nucleus or less stable when cytosolic. Nevertheless, a qualitative assessment of the overall spread of RNAscope signal throughout the section agrees with that of a higher vector dose in the 150% dose group.

## Discussion

There is little doubt that α-syn plays a key role in the pathogenesis of PD, perhaps participating in both cellular death and dysfunction. However, given that the exact mechanistic role of α-syn in PD etiopathology is unknown, there is a need for models that accurately emulate α-syn phenomena as they appear in disease. Although several α-syn-based models are actively used by the research community, none of these faithfully represent all aspects of the disease, and ultimately, research should be performed on multiple complementary models. To that end, the AAV-α-syn model serves an important purpose, providing researchers with a means to study α-syn pathology in a cell-autonomous manner, as transduction with standard AAV capsids is largely confined to neurons (Figure 4), and to assess systemic consequences (e.g., inflammatory responses) associated with such pathology. Nevertheless, this model is associated with high variability, with reports ranging from near complete nigrostriatal denervation to little toxicity at all^29^; results that likely stem from the large number of experimental permutations that can be achieved when considering various AAV components such as capsids and recombinant genetic elements. To that end, the ultimate goal of the current study was to empirically determine which key components of this model, specifically genomic structure, capsid, and promoter, would provide the most robust means to achieve neurodegeneration. Moreover, we aimed to validate key outcome measures by testing several production batches, as well as performing complementary experimentation at two locations. These efforts were aimed at making this model as reproducible and accessible as possible to the general research community.

### Dose dependence and progression

In our initial triage, we arrived at scAAV5-CBh-α-syn as our lead vector. AAV5, although perhaps not the most potent neurotropic vector, is reported to have high affinity for dopamine neurons.^34,60,61^ A self-complementary genome bypasses the rate-limiting step in the infectious cycle, the generation of a double-stranded genome, which can either occur through annealing of + and – sense genomes or by a second-strand synthesis, thus, effectively reducing the needed MOI whilst maintaining expression levels.^62^ Finally, the CBh promoter is well-established to provide robust and persistent expression in post-mitotic cells such as neurons.^63^ As has been shown, we also observed relatively specific nigrostriatal denervation (i.e., the VTA was spared) that was both dose-dependent and progressive, with the concomitant S129 phosphorylation and motor dysfunction. Importantly, we repeated many experiments at two sites and observed significant concordance between our results.

### Problems with control vectors

One would be amiss not to discuss the issues that we observed with controls. It is a well-known fact that fluorophores such as GFP and BFP are neurotoxic in vulnerable neuronal populations.^16,40–42^ Although the exact source of toxicity of these proteins is unknown, oxidative stress,^64^ heat-shock response,^65^ and cell-cycle dysregulation^66^ have been reported, with the globular nature of GFP and potential aggregation potentially playing a central role. Finally, these ectopic fluorophores are much larger in size than α-syn, possibly playing a role. Moreover, it has been well established that midbrain nigral neurons are particularly vulnerable to seemingly benign manipulations (reviewed in^67^), begging the question of how one best controls for α-syn overexpression. Studies have often manipulated variables that yield the best differential between treatment and control (e.g., lowering the titer of both the GFP control vector and the α-syn vector).^68^ Although this certainly is an experimentally appropriate control, it is, however, less than an ideal approach as this typically results in lower doses and lesions in the treatment group. Such adjustments can surely complicate studies in which one looks for a therapeutic effect, as one can only achieve a very small therapeutic window in terms of a rescue-effect. Finally, as we note, given the high discrepancy in various older titering methods, historical data regarding controls should be viewed with some caution.

In the current study, we ultimately arrived at a null-vector which controls for many of the aspects of AAV infection: mechanical disruption, extracellular exposure to capsid, receptor occupation, nuclear translocation, expulsion of capsid, and transcriptional activity, etc. Although one can argue that the lack of protein production makes this a less-than-ideal control, the fact that we indeed observe a complete lack of nigral cell loss is an important fact that cannot be ignored. It is also noteworthy that even the NV was not inert at higher doses (i.e., 150%) as we did observe some gliosis in this treatment group. Ultimately, the choice of control comes down to the experimental question at hand.^67^

#### The strengths of the AAV-α-syn model

The rationale for this study was to generate a strong foundation by enabling distribution of a validated research tool for the broader PD research community, with a focus on rigor and reproducibility. Whereas we did not emphasize a thorough characterization of molecular consequences following α-syn overexpression, we expect, in line with a large body of data, that reported etiopathological features, such as proteinase K resistant, thioflavin S, and ubiquitin +, α-syn aggregates, etc. would be present. We also expect additional behavioral phenotypes not assayed herein.^35,36^

The need for this validated research tool came from a firm belief that this model is an important component in PD *in vivo* models, with significant features lacking in other models. One chief advantage of the model lies in the approach itself, the use of AAV. This approach allows for manipulations that are not possible in other experimental systems or α-syn models. As we demonstrate, one can increase the dose (titer) of the vector and thereby both exacerbate and accelerate neurodegeneration. Conversely, the dose can be lowered to a point where there is no neurodegeneration. The flexibility of dosing is an important aspect that allows an experimenter to, for example, assess a therapeutic in different severity conditions or study peritoxic events, respectively. Moreover, the combination of a CRE-dependent genome (aka FLEX or DIO^69^) with CRE-expressing animals allows a researcher to guide α-syn expression and pathology to specific circuits. With the emergence of novel, engineered AAV capsids, the AAVs can also target specific subpopulations of the midbrain dopamine system based on the projection pattern^70–72^ or delivered intrathecally or systemically to model widespread synucleinopathy.

### Important methodologies

With this study, we also validated the use of AI based on convolutional neural network algorithms with supervised learning to assess nigral neurodegeneration in the AAV-α-syn model. Importantly, we validated the AI-model using conventional stereology and we also performed independent supervised learning approaches at the different sites. In both cases, we observed a very high concordance of the enumeration of TH + cells; these algorithms are available for public use on the AIforia platform.

An important aspect our study highlighted is the need for QC when using AAV, especially in uses such as the α-syn model, where the effective dose is essential in accurately predicting an outcome (i.e., extent of neurodegeneration). We consistently found that reported vector titers differed significantly from what we measured at both sites using either qPCR or ddPCR, and found ddPCR to be the most uniform measurement with much less variability (Table 1). Although the use of ddPCR as a titering method is growing, given that we observed discrepancies as high as 200% between titering methods, one needs to pay close attention to titering methodology when assessing historical data from this model.

In summary, based primarily on a readout of nigral neuron survival in the current set of experiments, we empirically chose a construct carrying a self-complementary AAV cassette with the CBh promoter controlling the expression of human α-syn, packaged into the AAV5 serotype. We validated the efficacy of this construct across batches, time points, and concentrations, establishing key dose ranges resulting in measurable neurodegeneration. These results lay the foundation for researchers who wish to use this construct in future PD research.

## Supplemental Material

sj-docx-1-pkn-10.1177_1877718X261447105 - Supplemental material for Construct optimization for AAV-mediated human α-syn overexpression, and validation across research settings: Development of a shared tool for the research community Supplemental material, sj-docx-1-pkn-10.1177_1877718X261447105 for Construct optimization for AAV-mediated human α-syn overexpression, and validation across research settings: Development of a shared tool for the research community by Joaquin Pardo, Janitha Mudannayake, Luis Daniel Bernal-Conde, Ivette M. Sandoval, Ryan B. Dong, Martino Avallone, David J. Marmion, Addison Gralen, Lauren Waite, Andreas Heuer, Marcus Davidsson, Nicole K. Polinski, Tomas Björklund and Fredric P. Manfredsson in Journal of Parkinson's Disease
