# Supplementary material for: Construct optimization for AAV-mediated human α-syn overexpression, and validation across research settings: Development of a shared tool for the research community
Source: J Parkinsons Dis. 2026 May 25:1877718X261447105. Online ahead of print. doi: 10.1177/1877718X261447105 (PMC13435177; doi:10.1177/1877718X261447105)
Supplement: Supplementary file 1 [file sj-docx-1-pkn-10.1177_1877718X261447105.docx]

**Supplemental Figure 1. Representative images showing AI-based detection and enumeration of TH+ cells.** A rat midbrain section is displayed at low and higher magnification. Somata of TH+ cells are quantified within selected ROIs by a CNN algorithm. *Images from the Aiforia Platform.*

**Supplemental Figure 2. *ISH*-based detection of transcripts in Experiment 4.** In order to validate the functionality of the null vector, we performed RNAscope *ISH* against the WPRE and performed densitometric quantitation of staining intensity. **A**. Interestingly the 100% α-syn group showed higher WPRE abundance compared to either NV group. Representative RNAscope images from α-syn 100% (**B**), NV 100% (**C**), and NV 150% (**D**). B’, C’, and D’ shows a higher magnification of area outlined in B, C, and D, respectively. * p < 0.05; 1-way ANOVA. Scalebar in D = 1.0mm and applies to B-D. Scalebar in D’ = 0.1mm and applies to B’-D’.
